# Mitochondrial ATPase activity and membrane fluidity changes in rat liver in response to intoxication with Buckthorn (*Karwinskia humboldtiana*)

**DOI:** 10.1186/s40659-015-0008-9

**Published:** 2015-03-19

**Authors:** Margarita Cid-Hernández, Ana C Ramírez-Anguiano, Genaro G Ortiz, Eddic W Morales-Sánchez, Luis J González-Ortiz, Sandra F Velasco-Ramírez, Fermín P Pacheco-Moisés

**Affiliations:** Departamento de Química, Centro Universitario de Ciencias Exactas e Ingenierías, Universidad de Guadalajara, Blvd. Marcelino García Barragán 1421, 44430 Guadalajara, Jalisco Mexico; División de Neurociencias, Laboratorio de Desarrollo, Envejecimiento y Enfermedades Neurodegenerativas, Centro de Investigación Biomédica de Occidente (CIBO), Instituto Mexicano del Seguro Social (IMSS), Sierra Mojada 800, 44340 Guadalajara, Jalisco Mexico

**Keywords:** *Karwinskia humboldtiana*, ATPase, Mitochondria, Membrane fluidity, pH gradient

## Abstract

**Background:**

*Karwinskia humboldtiana* (Kh) is a poisonous plant of the rhamnacea family. To elucidate some of the subcellular effects of Kh toxicity, membrane fluidity and ATPase activities as hydrolytic and as proton-pumping activity were assessed in rat liver submitochondrial particles. Rats were randomly assigned into control non-treated group and groups that received 1, 1.5 and 2 g/Kg body weight of dry powder of Kh fruit, respectively. Rats were euthanized at day 1 and 7 after treatment.

**Results:**

Rats under Kh treatment at all dose levels tested, does not developed any neurologic symptoms. However, we detected alterations in membrane fluidity and ATPase activity. Lower dose of Kh on day 1 after treatment induced higher mitochondrial membrane fluidity than control group. This change was strongly correlated with increased ATPase activity and pH gradient driven by ATP hydrolysis. On the other hand, membrane fluidity was hardly affected on day 7 after treatment with Kh. Surprisingly, the pH gradient driven by ATPase activity was significantly higher than controls despite an diminution of the hydrolytic activity of ATPase.

**Conclusions:**

The changes in ATPase activity and pH gradient driven by ATPase activity suggest an adaptive condition whereby the fluidity of the membrane is altered.

## Background

*Karwinskia humboldtiana* currently known by the common names buckthorn, wild cherry, tullidora, capulin tullidor, capulincillo, coyotillo, and cacatsin, is a poisonous shrub of the Rhamnaceae family that grows from Southern United States to northern Colombia [1]. Medicinal properties have been attributed to several species of the genus *Karwisnkia* due to a secondary metabolite with antineoplastic action on mammalian tumor cells [2].

The ingestion of the green or ripe fruit of Kh causes a flaccid, symmetric, progressive, and ascending palsy of the lower limbs, which, in severe cases may progress to quadriplegia and breathing insufficiency, with victims requiring assisted mechanical ventilation. The neurologic symptoms are similar to those of poliomyelitis, Guillain-Barre syndrome, and other polyradiculoneuritis syndromes, for which it is often mistaken. In Mexico, the accidental ingestion of Kh causes several health problems [3-5], particularly there are numerous reports of poisoning by Kh, of which 68.1% of cases occurred in children under 5 years old [5]. Kh contains significant amounts of phenolic antracenonas and/or anthraquinone [3,6].

Oral administration of a slurry prepared with the seed of Kh in rats causes a uncoordinated motor activity of the hind limbs followed by rapidly ascending flaccid paralysis and signs of respiratory distress [7]. A histological study showed lesions in peripheral nerves corresponding to a segmental demyelinating neuropathy and specific lesions in the cerebellum, cerebral cortex, reticular formation, possibly due to anoxia [8]. Deaths in cats have been reported after acute administration of higher doses [9]. Paralysis caused by Kh is due to blockage in the conduction of nerve impulses, and to muscle denervation [7]. Other authors have reported damage in organs such as liver (necrosis and fatty degeneration), lung (pulmonary hemorrhage), heart (necrosis) and kidney [10] Furthermore a decrease of peroxisomes has been reported in rat hepatocytes [11].

The structure and function of cell membranes plays an important role in maintaining intracellular homeostasis through the activity of membrane enzymes, hormone receptors, and the transmembrane transport system. These dynamic phenomena are influenced by membrane fluidity. Fluidity is an important characteristic of biologic membranes at a molecular level. It indicates the way and the rate of motion of molecules in the membrane, and is inversely related to membrane microviscosity. Changes in membrane fluidity may play a role in the regulation of membrane properties, both under normal and pathological conditions [12]. Mitochondria provide cellular energy via oxidative phosphorylation, using the multisubunit complexes of the respiratory chain to create a transmembrane potential, which drives ATP synthesis by the ATP synthase; ATP synthase is also capable of working as an ATPase [13].

It has been reported that mice fed with Kh showed some hepatic changes such as degeneration and necrosis, in addition to neurologic disease and weight loss [14]. Furthermore, a single dose of an oral preparation from the seed fruits of Kh causes a significant decrease of ATP concentrations in renal tissue [10] and liver [15]. In mitochondria isolated from liver of rats, the petroleum ether extracts from Kh fruits, induces inhibitory and coupling effects on respiration and oxidative phosphorylation [16], suggesting that alterations on energetic metabolism and oxidative stress are related with the mechanism by which Kh induces its cytotoxic effects. These findings prompted us to investigate the effect of an extract of Kh seeds on membrane fluidity and mitochondrial ATPase activities in rat liver.

## Methods

### Plant material

Fruits were collected from August to October, 2010 at the settlements of Santa Cruz and Santa Gertrudis, municipality of Colotlán, state of Jalisco (México) and were dried at room temperature and light protected. Before its administration, the almond and cuticle of the seeds were removed, then ground, weighed and mixed with peanut butter to form a homogeneous paste (1:3, weight/weight).

T-514 anthracenone from the cuticle and almond of the seeds of *K. humboldtiana* were isolated and quantified by procedures already described [17]. The content of T-514 in the samples used was 20.5% of the total anthracenones. (1 g of tissues contains 0.13 g of toxin T-514).

### Experimental animals

Thirty five adult male Sprague Dawley rats (obtained from the animal facility of Centro de Investigación Biomédica de Occidente, Guadalajara, Jalisco, Mexico) weighing 250–300 g, maintained on a 12 h/12 h light–dark cycle at 25 ± 2°C, fed with standard diet (Chow Purina) and water ad libitum were used for this research work. All animals received humane care based on the international guidelines on the ethical use of animals, and the “Norma Oficial Mexicana NOM-062-ZOO-1999”. Experimental procedures were approved by the local animal ethics committee (Guadalajara, Jalisco, Mexico). Animals were randomly assigned into control non-treated group (n = 5) that received only vehicle (peanut butter) and three Kh treated groups (n = 10) that received 1, 1.5 and 2 g/Kg body weight of dry powder of Kh fruit, respectively. We take special care to ensure that all peanut butter was eaten by rats. Five rats of each group were euthanized at day 1 and 7 after treatment, respectively. Rats euthanized at day 7 received a second dose of Kh, 24 h after the first dose.

### Submitochondrial particles isolation

After treatment, rats were sacrificed by decapitation and livers were immediately dissected for mitochondria isolation. Liver was homogenized with a teflon-on-glass homogenizer Potter-Elvehjem in 35 ml cold buffer containing 250 mM sucrose, 10 mM 4-(2-hydroxyethyl)-1-piperazineethanesulfonic acid (pH 7.4), 1 mM ethylene glycol tetraacetic acid, and kept on ice. The homogenate was centrifuged at 600 *x*g for 5 min at 4°C. The pellet was discarded, and the supernatant was centrifuged at 8,000 *x*g for 10 min at 4°C. The foamy layer at the top of the supernatant was removed. The mitochondrial pellet was washed with buffer containing 0.1% fatty acid-free serum albumin and finally resuspended in buffer to contain about 20 mg/ml protein. Then, submitochondrial particles were obtained as previously described [18]. Membrane protein was measured by the method of Lowry *et al.* [19] using bovine serum albumin as standard.

### Membrane fluidity estimations

The degree of membrane lipid fluidity was quantitatively estimated from the excimer to monomer fluorescence intensity ratio (Ie/Im) of the fluorescent probe 1,3 dipyrenylpropane (DPP) incorporated in submitochondrial particles. Briefly, 0.25 mg of membranal protein and 0.1 nmol DPP were mixed with 10 mM Tris–HCl buffer (pH 7.8). The mixtures were incubated in darkness at 4°C for 3 hours, in order to achieve maximal incorporation of the fluorescent probe to the membranes. Fluorescence was measured at 30°C on a Perkin Elmer fluorescence spectrometer, LS50B. The fluorophore was excited at 329 nm and the monomer and excimer fluorescence intensities were read at 379 and 480 nm, respectively. From these readings, the Ie/Im was calculated. Fluorescence corrections obtained from readings of membranes without DPP were applied to all fluorescence values [20].

### ATPase activity quantification

Oligomycin-sensitive ATPase rates were assayed by the release of inorganic phosphate. The standard reaction medium (0.5 mL) contained 125 mM KCl, 40 mM Hepes/KOH (pH 8.0), 0.1 mM EGTA, 3 mM ATP, 5 mM MgCl_2_ and 2.2 mM oligomycin. The reaction was initiated by the addition of submitochondrial particles (0.25 mg of protein) [21]. It was quenched with 200 mL of cold 30% (w/v) trichloroacetic acid. Afterwards, the sample was centrifuged for 10 min at 3,500 rpm and 400 μL of the supernatant was separated, and 1 ml of 3.3% ammonium molybdate was added, followed by 100 μL of 10% ferrous sulphate. The reading of the absorbance of the samples was recorded at 660 nm [22] using the Benchmark Plus Microplate Spectrophotometer System, 110/230 V by Bio-Rad. These analyses were referred to phosphate curve for estimating the enzyme activity in submitochondrial particles. Control experiments showed that 2 μM oligomycin inhibits 90–95 of the ATPase activity in submitochondrial particles.

### pH gradient driven by ATPase

The pH gradient driven by mitochondrial ATPase activity was determined by the fluorescence quenching of 1 μM 9-amino-6-chloro-2-methoxyacridin (ACMA). ACMA fluorescent quenching is directly related to pH gradient, i.e., the higher values of fluorescent quenching, the higher values of pH gradient [23]. Briefly, submitochondrial particles (1 mg of protein) were incubated at 37°C in a medium (2 mL) containing 125 mM KCl, 20 mM MOPS (pH 7.5), 5 mM MgCl_2_, 0.1 mM EGTA, 1 μM ACMA, 3 mM ATP and 5 mM inorganic phosphate. After stabilization of the signal, membranes were energized with 3 mM ATP. ATPase activity pump protons into the interior of membranes, acidifying the internal space and the fluorescent amine were internalized. Therefore a substantial quenching of ACMA fluorescence is detected, indicating that a pH gradient (acidic inside) was established by the ATPase. In our experiments, addition of 1 nM of nigericin (H^+^/K^+^ exchanger) restored the signal, demonstrating that the pH gradient across the membranes resulted from the transport of protons.

### Statistical analysis

Results are expressed as the standard error of the mean (mean ± SEM). The significance of differences between groups was evaluated using One-way analysis of variance (ANOVA) and Bonferroni’s Multiple Comparison Test. Differences with P < 0.05 were regarded as significant.

## Results

**Figure 1:**
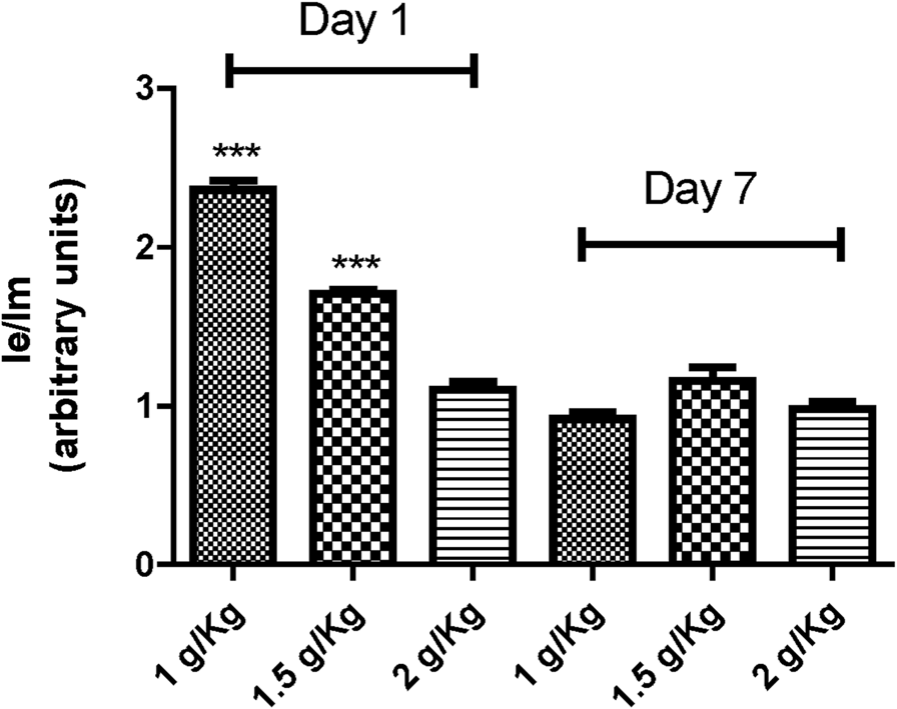
**Relative membrane fluidity in submitochondrial particles from rats subjected to the indicated dose of** ***Karwinskia humboldtiana*** **.** Relative membrane fluidity, as fold induction, was calculated by normalization of values to their respective controls (0.63 ± 0.04). Control value was fixed at 1.0. The bar indicates the mean ± S.E. ***indicate a significant difference from the control group (P < 0.001).

To assess the acute effects of Kh on membrane fluidity and ATPase activities, from each group, half of the rats were euthanized on day 1 and the remaining half at day 7 post Kh treatment. Figure 1 depicts the relative membrane fluidity of submitochondrial particles through the relative excimer/monomer (Ie/Im) ratio of DPP; the higher the Ie/Im ratio, the more fluid is the membrane. At day 1 of Kh treatment, the dose of 1 and 1.5 Kg/g increases significantly the fluidity of membranes relative to the control group. However, higher dose (2 g/Kg) does not modify substantially the fluidity of mitochondrial membranes. At day 7, membrane fluidity values in Kh-treated rats were similar to those of controls and no statistically significant differences were found.

**Figure 2:**
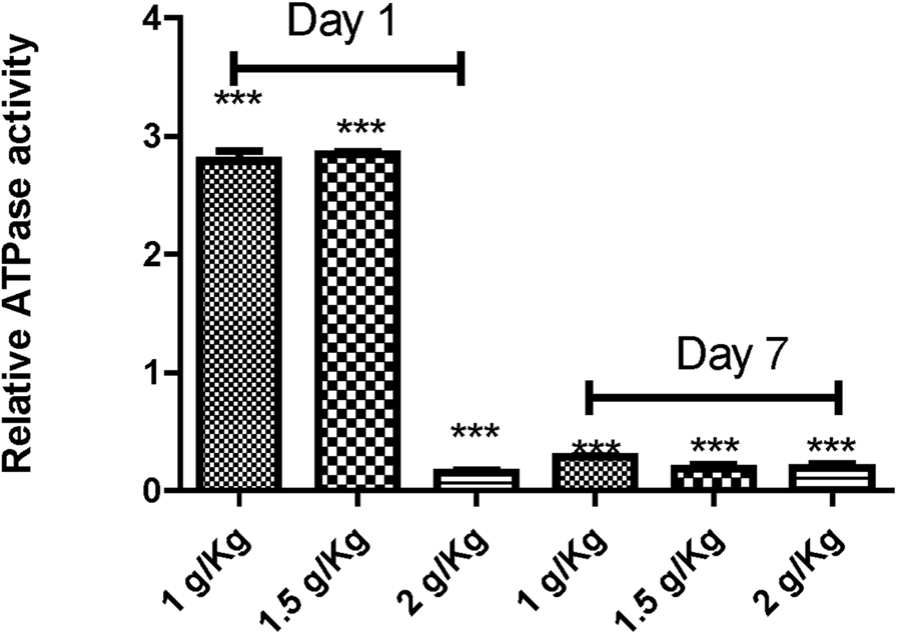
**Relative ATPase activity in submitochondrial particles from rats subjected to the indicated treatments.** Relative ATPase activity, as fold induction, was calculated by normalization of values to their respective controls (13.5 ± 1.43 nmol min^−1^ (mg protein^−1^). Control value was fixed at 1.0. The bar indicates the mean ± S.E. ***indicate a significant difference from the control group (P < 0.001).

Several major functions of membranes such as enzyme activities and ligand-receptor interactions depend on membrane fluidity. Therefore, to determine whether an alteration in enzymatic activity was related to changes in membrane fluidity, in this work we determined the ATPase activities in submitochondrial particles. A basal ATPase activity was detected in control rats. As shown in Figure 2, administration of Kh induced a significant increase of mitochondrial ATPase activity at the dose of 1 and 1.5 g/Kg, as compared to control rats, on day 1 after Kh treatment. Conversely, ATPase activity showed a significant reduction at day 1 when using a dose of 2 Kg/g, and day 7, at all doses.

**Figure 3:**
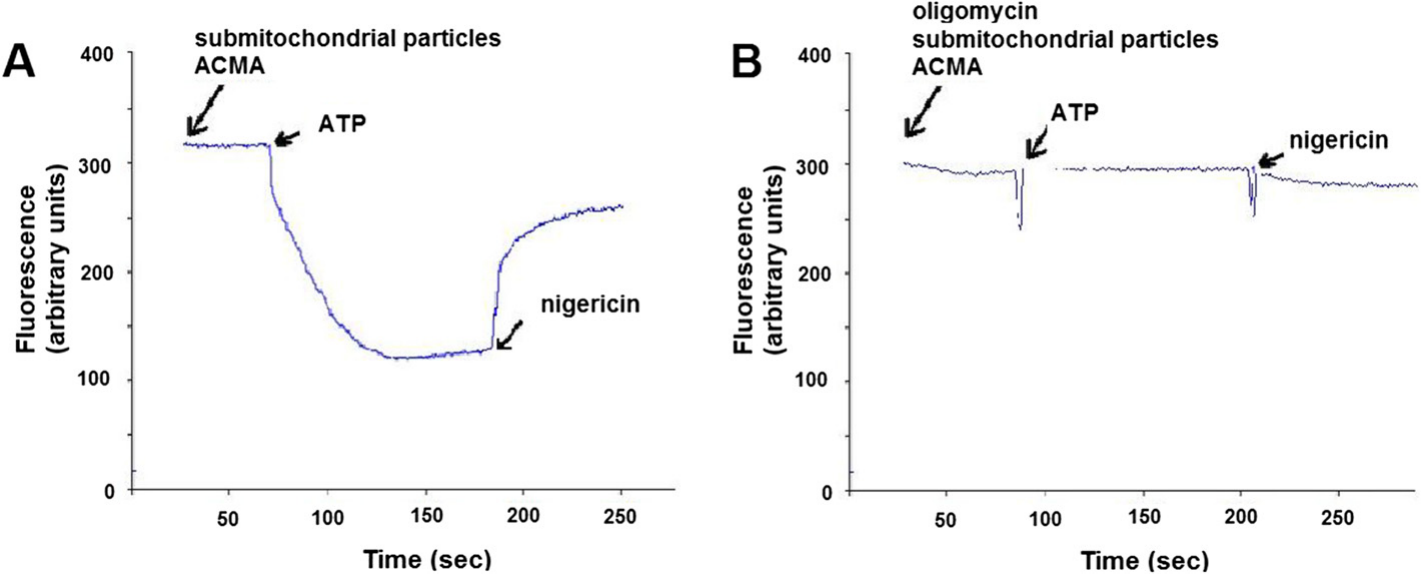
**ATP-driven quenching of ACMA fluorescence by submitochondrial particles from non-treated rats. (A),** The reaction was started by the addition of ATP and the fluorescence emission was recorded as indicated in Methods. **(B),** The submitochondrial particles were preincubated with 2 μM oligomycin and the reaction was started and recorded as in A.

**Figure 4:**
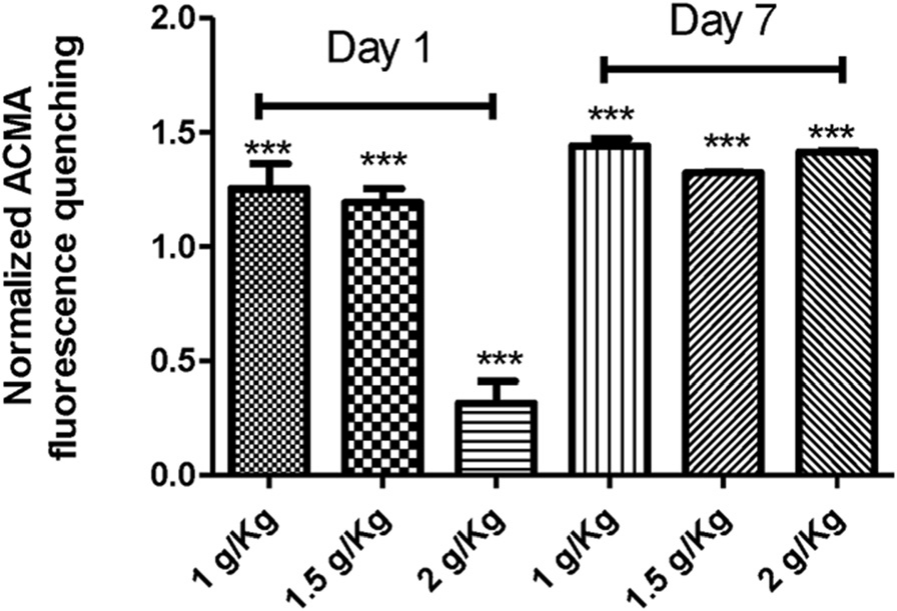
**Relative transmembrane pH gradient driven by ATP hydrolysis in submitochondrial particles from rats subjected to the indicated treatments.** Relative transmembrane pH gradient by ATP hydrolysis, as fold induction, was calculated by normalization of values to their respective controls (57.15 ± 5.81). Control value was fixed at 1.0. The bar indicates the mean ± S.E. ***indicate a significant difference from the control group (P < 0.001).

Transmembrane pH gradient driven by ATPase activity has been extensively used as indication marker of the correct function of the enzyme proton channel and coupling between transport and catalysis [21,24]. Control experiments showed that ACMA fluorescence quenching was not detected when membranes were incubated briefly with 2 μM oligomycin (Figure 3). A substantial pH gradient driven by ATPase activity was detected in liver submitochondrial particles from non-treated rats. In addition, Kh treatment induces a significant increase of pH gradient at lower doses on day 1 and conversely, at the higher dose tested induces a diminution of pH gradient. To our surprise, pH gradient was higher than control group, on day 7 post-treatment, at all doses tested (Figure 4).

## Discussion and conclusions

In this work we found that rats under Kh treatment at all dose tested does not developed any neurologic symptoms (not shown). However, we detected alterations in membrane fluidity and ATPase activity in liver submitochondrial particles. These changes were not linear over the dose-range studied. For instance, lower dose of Kh on day 1 after treatment induced higher membrane fluidity than control group. This change was strongly correlated with increases in the apparent ATPase activity and pH gradient driven by ATP hydrolysis. On the other hand, at the higher dose used of Kh extract (2 g/Kg) and day 1 post-treatment, there is no change of the membrane fluidity, but there is a decrease of ATPase activity and therefore a diminished pH gradient. Mitochondrial membrane fluidity was hardly affected on day 7 after treatment with Kh. In addition, the pH gradient driven by ATPase activity was significantly higher than controls despite a diminution of the hydrolytic activity of ATPase. These results suggest an adaptive condition whereby the fluidity of the membrane is altered. It has been proposed that changes in membrane fluidity can affect the conformation and activity of membrane-bound enzymes. However, in this work we found that the relationship between membrane fluidity and ATPase activity remains elusive, particularly at day 7 after Kh treatment.

It has been reported that the anthracenone T-514 isolated from *Karwinskia humboldtiana* showed a homogeneous distribution on subcellular fractions prepared from rat liver treated with an acute dose of T-514, indicating that T-514 can pass easily through sub cellular compartment membranes. In addition Kh treatment induced a significant increase of protein in liver homogenates and NADPH-cytochrome P450 reductase microsomal activity [25]. In consonance with these reported findings we showed that Kh treatment has specific effects on liver mitochondria and to our surprise Kh increases the pH gradient driven by ATPase activity.

The changes of mitochondrial membrane fluidity properties may have clinical relevance and can precede other alterations reported previously, such as the significant decrease of the number of peroxisomes present in the hepatocytes [26], the increased plasmatic activities of aspartate aminotransferase and alanine aminotransferase and decreased liver ATP concentration [15].

## References

[1] Knight AP, Walter RG, Knight AP, Walter RG (2001). Other plant affecting the nervous system. Guide to Plant Poisoning of Animals in North America.

[2] Waksman DE, Torres N, Santoyo A, Ramírez R, Piñeyro-López A (1997). Cuantificación de T514 (Peroxisomicina A1) en 2 plantas del género Karwisnkia. Polibotánica.

[3] Becerra-Verdin EM, Bermúdez-Barba MV, Salazar-Leal ME, Ancer Rodríguez J, Romero-Diaz V, Soto-Domínguez A (2009). Karwinskia humboldtiana (buckthorn) fruit causes central nervous system damage during chronic intoxication in the rat. Toxicon Off J Int Soc Toxinology.

[4] Charlton KM, Pierce KRA (1970). Neuropathy in Goats Caused by Experimental Coyotillo (Karwinskia humboldtiana) Poisoning : II. Lesions in the Peripheral Nervous System − Teased Fiber and Acid Phosphatase Studies. Pathol Vet.

[5] Nava Arreola EM, Vázquez Castellanos JL, González Castañeda ME (2000). Factores geográficos en la epidemiología de la intoxicación por Karwinskia (tullidora) en México. Cad Saude Publica.

[6] Garza-Ocañas L, Jiang T, Acosta D, Torres-Alanis O, Waksman De Torres N, Piñeyro-Lopez A (1994). Comparison of the hepatotoxicity of toxin T-514 of Karwinskia humboldtiana and its diastereoisomer in primary liver cell cultures. Toxicon Off J Int Soc Toxinology.

[7] Salazar-Leal ME, Flores MS, Sepulveda-Saavedra J, Romero-Diaz VJ, Becerra-Verdin EM, Tamez-Rodriguez VA (2006). An experimental model of peripheral neuropathy induced in rats by Karwinskia humboldtiana (buckthorn) fruit. Periphenal Nerv Syst.

[8] Ocampo-Roosens LV, Ontiveros-Nevares PG, Fernández-Lucio O (2007). Intoxication with buckthorn (Karwinskia humboldtiana): report of three siblings. Pediatr Dev Pathol.

[9] Waksman N, Martinez L, Fernandez R (1989). Chemical and toxicological screening in genues Karwinskia (Mexico). Rev Latinoam Química.

[10] Jaramillo-Juárez F, Rodríguez VML, Castillo CMG, Quezada TT, de la Cerda GE, Del Posadas RFA (2009). Daño hepático y en la coagulación de la sangre producido por la administración de los frutos maduros de la planta tullidora (Karwinskia humboldtiana) en la rata. Rev Mex Ciencias Farm.

[11] Sepúlveda-Saavedra J, Bermúdez De Rocha MV, Tamez-Rodríguez VA, Ballesteros-Elizondo RG, Moreno-Sepúlveda M, Piñeyro-López A (1998). Quantitative analysis of liver peroxisomes in rats intoxicated with peroxisomicine-A1. Toxicol Lett.

[12] Schuller A, Solis-Herruzo JA, Moscat J, Fernandez-Checa JC, Municio AM (1986). The fluidity of liver plasma membranes from patients with different types of liver injury. Hepatology.

[13] Boyer PD, The ATP (1997). Synthase-a splendid molecular machine. Annu Rev Biochem.

[14] Bermudez MV, Gonzalez-Spencer D, Guerrero M, Waksman N, Piñeyro A (1986). Experimental intoxication with fruit and purified toxins of buckthorn (Karwinskia humboldtiana). Toxicon Off J Int Soc Toxinology.

[15] Jaramillo-Juárez F, Rodríguez-Vázquez ML, Muñoz-Martínez J, Quezada-Tristán T, Posadas Del Río FA, Llamas-Viramontes J (2005). The ATP levels in kidneys and blood are mainly decreased by acute ingestion of tullidora (Karwinskia humboldtiana). Toxicon Off J Int Soc Toxinology.

[16] Wheeler MH, Camp BJ (1971). Inhibitory and uncoupling actions of extracts from Karwinskia humboldtiana on respiration and oxidative phosphorylation. Life Sci Pt 2 Biochem Gen. Mol Biol.

[17] Dreyer DL, Arai I, Bachman CD, Anderson WR, Smith RG, Daves GD (1975). Toxins causing noninflammatory paralytic neuronopathy. Isolation and structure elucidation. J Ame Chem Soc.

[18] Thayer WS, Rubin E (1979). Effects of chronic ethanol intoxication on oxidative phosphorylation in rat liver submitochondrial particles. J Biol Chem.

[19] Lowry OH, Rosebrough NJ, Farr AL, Randell RJ (1951). Protein measurement with the Folin phenol reagent. J Biol Chem.

[20] Ortiz GG, Pacheco-Moisés F, EL Hafidi M, Jiménez-Delgado A, Macías-Islas MA, Rosales Corral SA (2008). Detection of membrane fluidity in submitochondrial particles of platelets and erythrocyte membranes from Mexican patients with Alzheimer disease by intramolecular excimer formation of 1,3 dipyrenylpropane. Dis Markers.

[21] Martinez Cano E, Ortiz GG, Pacheco-Moises F, Macias Islas MA, Sanchez-Nieto S, Rosales Corral SA (2005). Functional disorders of F0F1-ATPase in submitochondrial particles obtained from platelets of patients with probable Alzheimer’s disease. Rev Neurol.

[22] Sumner JB (1964). A method for the colorimetric determination of phosphorus. Science.

[23] Rottenberg H, Moreno-Sanchez R (1993). The proton pumping activity of H + −ATPases: An improved fluorescence assay. Biochim Biophys Acta.

[24] Baracca A, Barogi S, Carelli V, Lenaz G, Solaini G (2000). Catalytic activities of mitochondrial ATP synthase in patients with mitochondrial DNA T8993G mutation in the ATPase 6 gene encoding subunit a. J Biol Chem.

[25] Guerrero-Olazarán M, Viader-Salvadó JM (1996). Natural anthracenone subcellular distribution and effects on NADPH-cytochrome P450 reductase microsomal activity. Drug Chem Toxicol.

[26] Vargas-Zapata R, Torres-González V, Sepúlveda-Saavedra J, Piñeyro-López A, Rechinger KB, Keizer-Gunnink I (1999). Peroxisomicine A1 (plant toxin-514) affects normal peroxisome assembly in the yeast Hansenula polymorpha. Toxicon.

